# Isolated pulmonary artery involvement in Takayasu arteritis: case report and review of the literature

**DOI:** 10.1186/s43044-023-00416-8

**Published:** 2023-10-10

**Authors:** Jie Li, Jun Xu, Ping Bao, Hongmei Li

**Affiliations:** 1 Department of Radiology, Northern Jiangsu People’s Hospital (Clinical Medical School of Yangzhou University), No.98, Nantong West Road, Yangzhou, 225001 China; 2 Department of Cardiac Function Examination, Northern Jiangsu People’s Hospital (Clinical Medical School of Yangzhou University), Yangzhou, Jiangsu China

**Keywords:** Takayasu arteritis, Pulmonary arteritis, Computed tomography angiography, Echocardiogram, Case report

## Abstract

**Background:**

Takayasu arteritis (TAK) is a chronic inflammatory arteritis. It most often affects non-specific large vessel progressively, such as the aorta and its branches. The diagnosis in TAK is typically delayed. Isolated pulmonary artery involvement in Takayasu arteritis is uncommon. Owing to its rarity, the diagnosis is challenging and requires an integrated approach comprising clinical and imaging findings. In order to facilitate early diagnosis of TAK for clinicians, wider use of non-invasive imaging is impacting this.

**Case presentation:**

In this report, we present the imaging characteristics of a rare isolated pulmonary artery involvement in Takayasu arteritis. Pulmonary computed tomography angiography revealed only limited thick walls of both proximal pulmonary artery and stenosis of pulmonary artery lumen, and the other large blood vessels were not involved. The patient undergone pulmonary endarterectomy and pulmonary angioplasty. Then, approximately one month afterward, she passed away due to heart failure.

**Conclusions:**

Imaging examination is the main basis for diagnosing this disease. This impression might improve disease awareness among doctors and progress in diagnosis.

## Background

Takayasu arteritis (TA) is a chronic progressive non-specific large vessel vasculitis. It involves the aorta and its large branches predominantly. It occurs generally in women of childbearing age [1]. Pulmonary hypertension (PH) occurs in 12 to 13% of patients with TA and in 42.2% of patients with TA-pulmonary artery involvement (TA-PAI) [2]. TA-PAI can be affected isolatedly. Isolated pulmonary artery involvement (PAI) is a rare condition that affects 5.7% to 36.7% of TA patients only.

## Case presentation

**Fig. 1:**
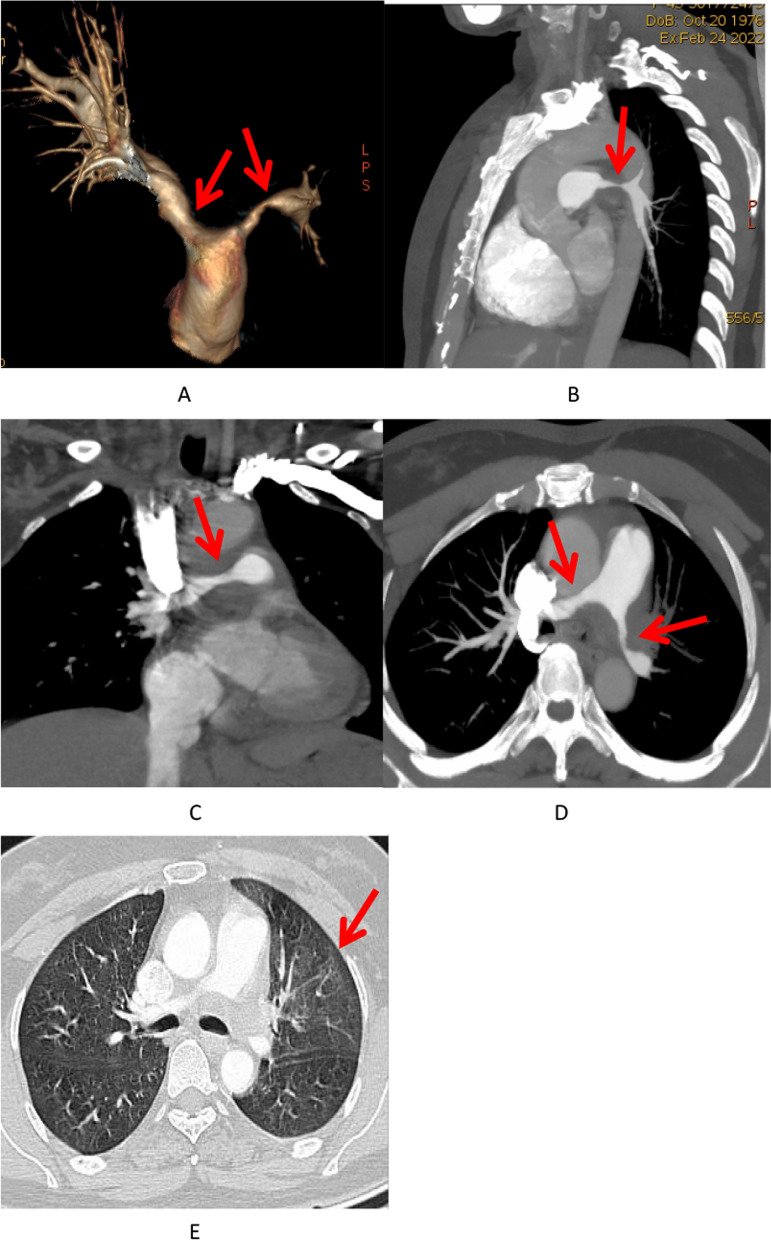
**A**–**D** Pulmonary computed tomography angiography (CTA) images. E HRCT. **A**, **D** showing thick walls of both main PA and stenosis of both main PA lumen (red arrows). **B** Display severe stenosis of the left PA (red arrows). **C** Showing mild-to-moderate stenosis of the right PA (red arrows). (**E**) Display the mosaic sign of the left lung (red arrows)

**Fig. 2:**
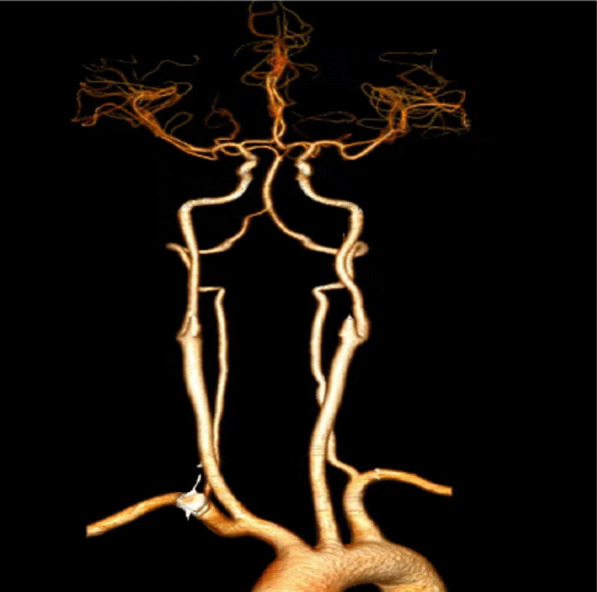
Computed tomography angiography (CTA) images showing unremarkable artery of bilateral subclavian, common carotid, internal carotid and vertebral arteries

**Fig. 3:**
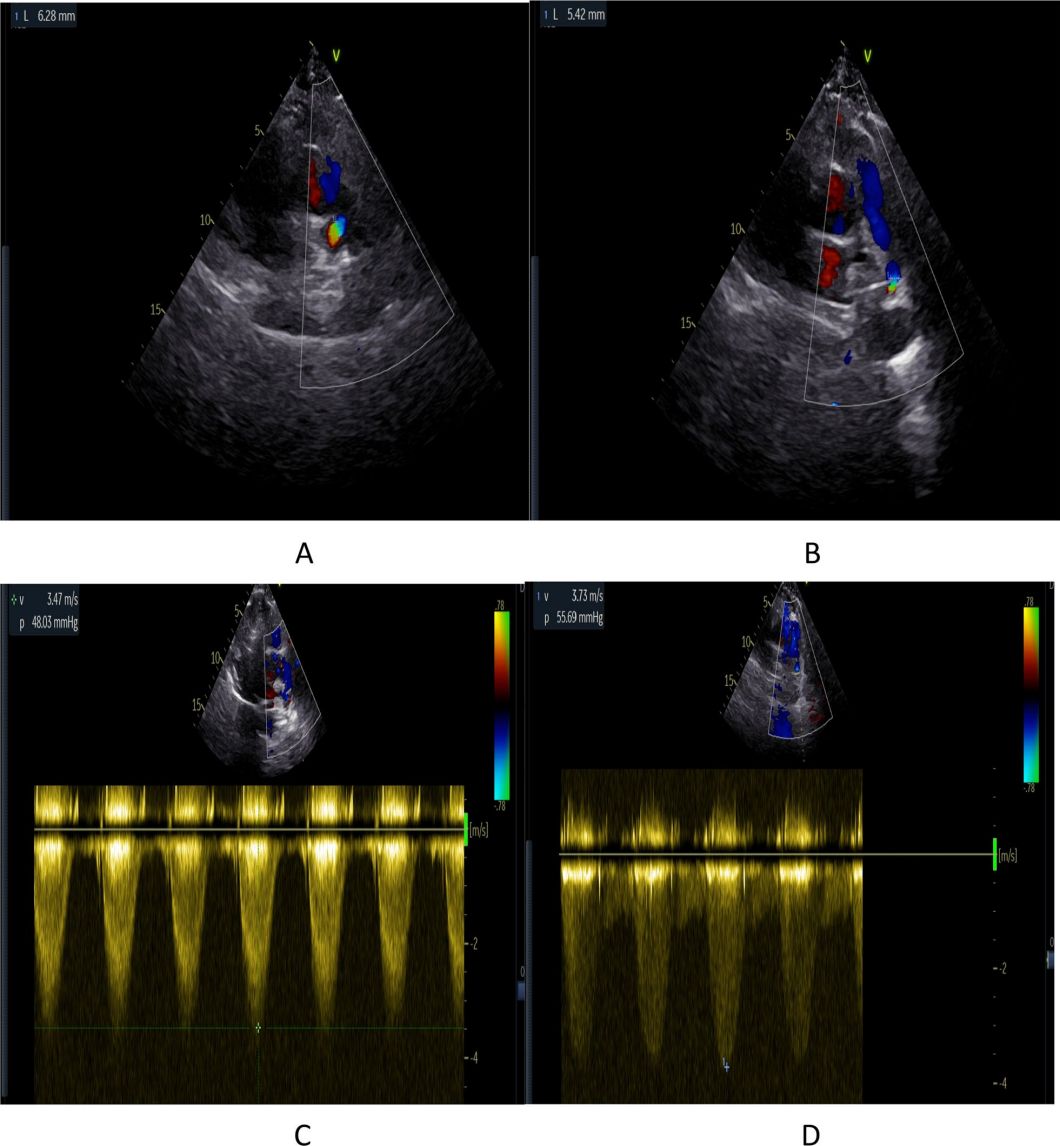
**A**–**D** Echocardiogram (**A**), **B** the narrowest diameter of right and left proximal PA. **C**, **D** The blood flow velocity and the maximum pressure difference of the right and left PA

**Fig. 4:**
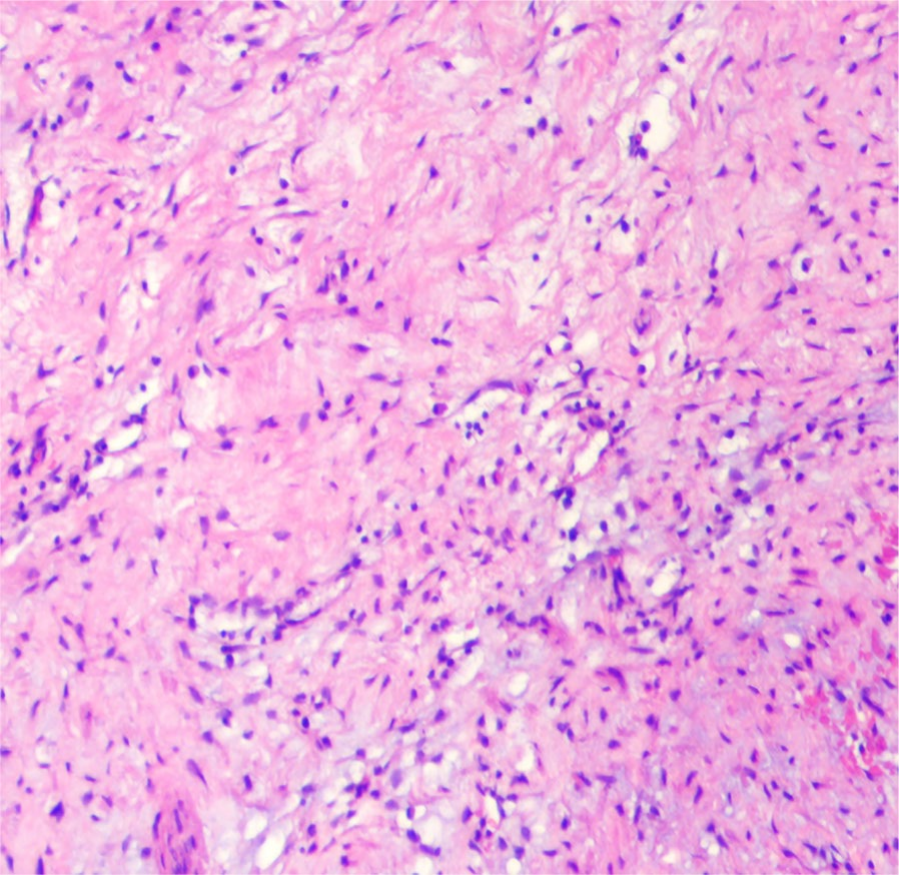
The hematoxylin and eosin-stained sample showing vitreous collagen fibers, mucoid degeneration and lymphocytes infiltration in the interstitium of pulmonary artery wall (× 100 multiple)

An 46-year-old congenitally deaf woman was presented to our hospital with chest tightness and edema of both lower extremities 5 days ago. Except for systolic ejection murmur in the auscultation area of pulmonary valve, her physical examination was unremarkable. The laboratory results indicated N-terminal pro-B-type natriuretic peptide of 1060.00 pg/ml (< 125 pg/ml), a D-dimer of 0.91 mg/L (< 0.55 mg/L), an erythrocyte sedimentation rate in first hour (ESR) of 60 mm/h (< 26 mm/h) and a C-reactive protein concentration (CRP) of 15.77 mg/dL (< 5 mg/dL). M. Tuberculosis interferon-γ release assay (TB-IGRA) and Treponema pallidum (TPPA) are negative. Electrolytes, infectious markers, and autoantibodies were normal. Pulmonary computed tomography angiography (CTA) was scanned on GE Light Speed VCT with injection 3.0 ml/s contrast. It revealed limited thick walls of both sides proximal pulmonary artery and severe stenosis of pulmonary artery lumen (Fig. 1A-D), with the normal images of pulmonary parenchymal. The left side pulmonary artery stenosis was even worse than the right side (5.4 mm vs. 6.3 mm). The images of the aortic vessel and head and neck were unremarkable (Fig. 2). On echocardiogram revealed stenosis of the left and right proximal pulmonary artery with narrowest diameter of 5.4 mm and 6.3 mm, respectively (Fig. 3A-B), with severe pulmonary hypertension about 100 mmHg. The maximum flow velocity of left and right pulmonary artery was about 3.7 m/s and 3.5 m/s. The maximum pressure difference of left and right pulmonary artery was about 55.7 mmHg and 48.0 mmHg (Fig. 3C-D). The patient undergone pulmonary endarterectomy and pulmonary angioplasty. Then, about a month later, she died of heart failure. Postoperative pathology assessment revealed vitreous collagen fibers, mucoid degeneration and inflammatory cell such as lymphocytes infiltration could be seen in the interstitium, which indicated Takayasu arteritis (Fig. 4).

## Discussion

CTA stands as the primary approach in diagnosing isolated PAI. The primary manifestation frequently observed was partial stenosis and occlusion of the pulmonary artery [3]. In a few cases, there were manifestations of pulmonary artery dilatation or pulmonary aneurysm [4]. It was often bilateral involvement. However, the right side was more often than the left in unilateral involvement cases [5].

The histological features of Takayasu arteritis are the presence of granulomatous inflammatory cell infiltration, destruction of smooth muscle and fibrosis in the media and adventitia. PH is a serious pulmonary circulation disease, usually with a worse prognosis. Pulmonary embolism can exhibit similar symptoms to isolated PAI at times.

Although both are manifested as pulmonary artery lumen stenosis and occlusion, pulmonary embolism involves mainly of the small-to-medium diameter pulmonary artery branches. The embolus lies in center of artery lumen, without enhancement. Isolated PAI with localized eccentric thick wall. It mostly involve from the main trunk to the branches, liking "rat tail". The wall is stiff and twisted with mild enhancement [6]. The final diagnosis is made by pathology. Glucocorticoids are the basic and effective treatment [7], and vascular surgery is also an important option for some severe cases [8].

## Conclusions

Isolated PAI is a relatively rare diseases that poses a diagnostic challenge. It is characterized by thickening of the pulmonary artery wall and stenosis of the pulmonary artery lumen, without involvement of other large blood vessels. Our cases impression might improve disease awareness among doctors and progress in diagnosis for isolated PAI.

## Data Availability

Not applicable.

## Abbreviations

TA: Takayasu arteritis
PA: Pulmonary arteritis.
CTA: Computed tomography angiography
HRCT: High-resolution computed tomography
PAI: Pulmonary artery involvement
PH: Pulmonary hypertension
CRP: C-reactive protein concentration
ESR: Erythrocyte sedimentation rate in first hour
